# A Schistosomiasis Research Agenda

**DOI:** 10.1371/journal.pntd.0000032

**Published:** 2007-12-26

**Authors:** Daniel G. Colley, W. Evan Secor

**Affiliations:** 1 Center for Tropical and Emerging Global Diseases and Department of Microbiology, University of Georgia, Athens, Georgia, United States of America; 2 Division of Parasitic Diseases, Centers for Disease Control and Prevention, Public Health Service, Atlanta, Georgia, United States of America; Swiss Tropical Institute, Switzerland

## Abstract

There is a long and rich history of research and control in the field of schistosomiasis that has resulted in major scientific and public health accomplishments. Examples of such findings and accomplishments include immunologic regulation in chronic infections [1], the association of helminth infections with Th1-regulating Th2-type immune responses [2], the critical role of interleukin-13 in fibrogenesis [3], and the development and validation of the “dose pole” for determining praziquantel dosages in the field [4],[5]. Perhaps in part because of this broad and successful history, those who work on schistosomiasis come from a wide variety of backgrounds and interests. While such variety is enriching to the field, it sometimes results in diverse opinions about which of the many research opportunities should be pursued. Such diversity, we believe, has at times led to a divisiveness that has harmed overall progress in the field. Partly in response to such events, we have worked with as many of those interested in schistosomiasis as we could identify to develop what we feel is a comprehensive and cohesive agenda for schistosomiasis research (Image 1).

## The Need for a New Research Agenda

We did not develop such an agenda as an attempt to work around or displace other efforts to organize schistosomiasis-related programs (for example, existing research or control networks or the current agenda set by the World Health Organization [WHO] or the UNICEF/UNDP/World Bank/WHO Special Programme on Research and Training in Tropical Diseases [TDR]). In fact, this agenda was developed with full knowledge and input from some of these existing programs. Nor is this agenda a means to obtain funding or to provide a priority listing of what kind of schistosomiasis research is needed to achieve given, spelled-out objectives, although we hope that the agenda may eventually be used to further both of these goals. Rather, we initiated this effort to help advance schistosomiasis research by enhancing cooperation and communication among the community of investigators interested in this neglected tropical disease, with the eventual goal of making stronger contributions to both biomedical science and public health.

## Origins and Development

The possible development of a comprehensive schistosomiasis research agenda was first discussed in a symposium at the annual meeting of the American Society of Tropical Medicine and Hygiene in Washington, D. C., in December 2005. This symposium actually followed by a month a meeting of the WHO/TDR Scientific Working Group (SWG) on Schistosomiasis, held in Geneva in November 2005. Both coordinators of the current agenda participated fully in the SWG on Schistosomiasis, and we strongly encourage interested readers to read the recently published proceedings from that meeting of 63 investigators and public health officials [6]. Following the American Society of Tropical Medicine and Hygiene symposium, the two of us then wrote an initial draft based on e-mail-solicited input received from first 22, then 110, and eventually over 150 people in the field. From January 2006 until now a total of about 350 people involved in schistosomiasis-related work were asked by e-mail for their input. We also presented the draft agenda for discussion in two open fora, first at the XIth International Congress of Parasitology Associations in Glasgow, Scotland (August 2006), and then at the American Society of Tropical Medicine and Hygiene meeting in Atlanta, Georgia (November 2006).

The final consensus schistosomiasis research agenda is provided in full in Boxes 1 - 4. The agenda will hopefully be a useful document to those from across the complete spectrum of the schistosomiasis community, from very basic research to focused and effective public health intervention. We also hope that as a result of feedback from the community about the agenda, the document itself will evolve over time.

## How Can the Agenda Be Used?

From one perspective, the new agenda may appear to be nothing more than an exhaustive “laundry list” of every type of study needed on schistosomiasis. The agenda also has elements that could be applied to almost any neglected tropical disease. Nevertheless, almost the entire schistosomiasis community participated in its development, trying to make the distinct parts fit a united whole. The agenda is not an attempt to prioritize one discipline of schistosomiasis research over another. We specifically avoided doing this because it is unlikely that any one funding agency would be interested in programs across the entire spectrum. In addition, we believe that attempts to prioritize specific areas of research from this broad agenda would prove to be unproductive and would create unnecessary factions. However, it may be valuable for researchers within one arena—for example, vaccine development or transmission dynamics—to use the agenda to prioritize research needs within their own field. It may also be useful for a group of individuals to take components from different sections of the agenda that fit well together and then approach funding agencies with linked components from the agenda. For example, a group of investigators who wanted to work together on development of a new, more sensitive, robust, field-applicable assay for active infection could merge relevant aspects of the agenda into a plan that spans genomics through field testing, including community acceptability, and then take that plan forward to a funding agency. If the consensus agenda is utilized in developing the proposal, both the group of investigators and the funding agency would be reassured that its foundation arose from the group effort of literally hundreds of experts in the field.

## How the Agenda Will Evolve Over Time

The agenda is not intended to be a static document. Rather, if it is to be relevant to the community, topics and approaches must be added and removed as new discoveries are made. The overarching goal of the agenda is to be inclusive of all those aspects of schistosomiasis-related research that are considered by the schistosomiasis community to be worthwhile, from both a basic scientific perspective and, obviously, in relationship to ultimate disease and infection control. We also hope to work with the Public Library of Science (PLoS) to create a schistosomiasis community portal based upon the online functionality in the recently launched *PLoS ONE* journal (http://www.plosone.org/), in which readers can annotate the literature, start discussion threads, and upload their own editorial commentaries. Through such an interactive process of community interchange, the new schistosomiasis research agenda can be continually commented upon, rearranged, and rewritten, as needs be.

**Box 1. Tools and Interventions**

A. Drugs

1. Optimization of treatment regimens in different transmission conditions
2.
  - Number of doses
  - Dose intervals
3. Mechanisms of action (old and new drugs)
4. Identification of schistosome proteins/pathways that are candidates for drug action
5. Development and testing of new drugs (e.g., orally active ozonides)
6.
  - Development of schistosome cell lines for high-throughput screening
  - Functional expression of putative drug targets
  - RNAi analysis for the detection of target molecules
7. Assays for standardization of drug quality
8. Development and standardization of assays and markers for resistance to praziquantel
9. Monitoring the nature and spread of drug resistance, and its effect on schistosomes
10. Combinatorial effects of anti-schistosome therapies
11.
  - Artemisinin-based combination therapies
  - Combinations of established anti-schistosome drugs with new drugs as registered
12. Pharmacokinetics
13.
  - Effects of infections and coinfections
  - Food intake
  - Intensity of infection and transmission
14. Impediments to treatment
15.
  - Access to drugs
  - Access to other health care services
  - Access to appropriate information, education, and communication for infected communities
16. How do real and/or perceived adverse events affect control programs?

B. Diagnostics

1. Optimization and combination of existing tools (immunological, ultrasound)
2. Assays for worm burden
3.
  - Sensitive, specific, inexpensive, field applicable, using accessible specimens
  -
    - High prevalence areas
    - Low prevalence areas
  - Able to distinguish active infection and successful cure
  - Investigate metabolites and other products as markers of infection
4. Tools for detection of morbidity or pre-morbidity
5. Validation of diagnostic approaches
6.
  - Central standardization
  - Uniformity of assays among studies and control programs
7. Surveillance tools for control programs
8.
  - Development and standardization of molecular monitoring
  -
    - Humans
    - Snails
  - Assessment of treatment failures
9. Sociocultural and economic factors influencing the validity of diagnostic tests

C. Control and implementation

1. Combined approaches to control
2.
  - Evaluate integrated use of treatment, sanitation, water supply, molluscicides, health communication, biological and environmental interventions, and eventually vaccines in combinatorial ways, and their community acceptability; develop comprehensive mathematical models incorporating these control measures and their clinical and economic impact
  - Evaluate integrated control programs, their efficacy and effectiveness
  -
    - School-based
    - Community-based
    - Combination of school- and community-based approaches
3. Treatment of special populations
4.
  - Use during pregnancy and lactation periods
  - Use during early childhood
5. Social aspects of control
6.
  - Health communications/education programs
  -
    - Evaluate combinations of content, communication means, participants and institutional settings adapted to local conditions
    - Perceptions, attitudes, and practices—the knowing and doing gap
  - Community involvement in control
  -
    - Social dynamics of snail control by environmental modifications
    - What are the incentives and disincentives at individual, household, village, and regional levels for praziquantel treatment and snail habitat modifications?
    - Evaluation to improve sustainability, including areas of low endemnicity
  - Control in health systems and inter-sectorial perspective
  -
    - What determines the cost-effectiveness of various control measures?
    - Enhancement of water resources development projects
    - What determines whether control is part of an integrated program?
    - Integration of control into community-directed treatment schemes
    - Dynamics of control as part of the broader health systems perspective (priority setting, resource allocation, public/private)

**Box 2. Ecological, Biological, and Societal Aspects of Transmission**

A. Hosts

1. Human
2.
  - Genetic studies
  - Age effects
3. Reservoir hosts of human species
4. Snails
5.
  - Tools to assess cercarial species, presence and release
  - Tools to identify and distinguish closely related snail species
  - Quantification and factors affecting absolute density of infected snails
  - Surveillance of immigrant snails into new areas
  - Assessment of genetic inbreeding on parasite transmission
  - Consequences of parasite coinfections in snails
  - Environmental impacts on parasite transmission to snails
  - Evaluation of new molluscicides
6. Genetic studies on host–parasite strain interactions and compatibility, including genomics, mathematical models, and population structure
7. Comparison of field versus laboratory parasite isolates

B. Fresh water

1. Positive/negative effects of pollution on snails and transmission
2. Impact of environmental change (dams, irrigation projects, etc.)
3. Development, implementation, use, and impact of appropriate technologies for water supplies
4. Environmental impact and effectiveness of molluscicide-based control
5. Impact of natural and exotic species on snails and on transmission

C. Transmission dynamics (including mathematical models)

1. Human
2. Natural, non-human hosts
3. Urban transmission
4. Low transmission areas after control programs
5. Social determinants of exposure (gender, ethnicity, occupation, migration)
6. Perceptions, attitudes, and practices—relationship to changes in transmission

D. Public awareness

1. Campaigns based on realistic DALYs (disability-adjusted life years) and impact of schistosomiasis to increase awareness and need (public, celebrity, politically based; at the local, national, regional, and international levels)

E. Application of geographic information systems/remote sensing and ground verification

1. Transmission patterns and predictions
2. Geo-spatial (micro) determinants of risk

**Box 3. Disease Burden and Epidemiology**

A. Morbidity

1. Attributable fraction
2.
  - Anemia and mechanisms of anemia
  - Under-nutrition
  - Organ dysfunction
  - Cognitive development
  - Economic costs of infection
3. Adapt standardized tools for quality of life assessment to schistosomiasis
4. Accurate disability weights, recalibration of DALYs
5. Impact of disease on households, communities, and societies
6. Carcinogenesis (with a focus on *Schistosoma haematobium* and possibly *S. japonicum*)
7. Effect of treatment on control of:
8.
  - Established morbidity (e.g., organomegaly, gynecological lesions, anemia, etc.)
  - Morbidity following reinfection
9. Effects on reproductive health and fertility (male and female)
10. Effects of host genetics

B. Comorbidities

1. Interactions of other infections with schistosomiasis (HIV, malaria, hepatitis B and C, soil-transmitted helminths)
2.
  - Effects of schistosomiasis and its treatment on coinfections
  - Effects of coinfections and their treatment on schistosomiasis
  - Effects of schistosomiasis on transmission of coinfections
3. Interactions and impact of dual schistosome infections *(S. mansoni and S. haematobium)*
4. Interactions of schistosomiasis with noninfectious conditions (malnutrition, alcoholism, autoimmunity)
5. Effects of schistosomiasis on vaccination programs

C. Pregnancy

1. Influence on child
2.
  - Morbidity
  -
    - In child in utero (e.g., low birth weight)
    - In child subsequently infected
  - Immunology
  -
    - Effect on neonatal vaccinations
    - If child subsequently infected
3. Treatment issues
4.
  - Need retrospective and prospective studies
  - Implementation, policy changes

**Box 4. Basic Science of Relevance to Schistosomiasis**

A. Vaccines

1. Discovery
2.
  - Antigens and protective responses in model and human systems
  - High-throughput vaccine design
  - Effects on infected or previously treated hosts (protective, pathologic, integration of vaccination with chemotherapy)
  - Vaccine types other than prophylactic (therapeutic, anti-fecundity)
  - Scale-up production (good laboratory/manufacturing practices)
  - Adjuvants/delivery (DNA versus prime boost versus protein)
3. Evaluation
4.
  - Model systems
  -
    - Closer look at animals that develop “sterile immunity”
    - Rapid assessment of vaccine efficacy
    - Non-human primates (interface of screening and clinical trials)
  - In the field (trial design, locales, end-points, interaction with other infections/vaccines, effect of prenatal exposures)

B. Immunology and pathology

1. During infection (human and model systems)
2.
  - Resistance versus susceptibility—mechanisms
  - Immunopathologic mechanisms
  -
    - Fibrosis
    - Angiogenesis
  - Immunoregulatory mechanisms
  - Host responses to defined antigens
  - Immune evasion
  - Effects on non-immune systems (e.g., hematologic, coagulation, pharmacologic)
3. Effects on immune response system
4.
  - Innate immune alterations/identification of schistosome pathogen-associated molecular patterns
  - Atopic allergy/role of schistosomiasis in “hygiene hypothesis”
  - Autoimmune diseases
  - Schistosome molecules as adjuvants
5. Role of host genetic polymorphisms (resistance and morbidity)
6. Effect of treatment on immune responses
7. Snail responses to infection

C. Genomes and postgenomics (of parasite life-cycle forms and snail species; in situ and ex vivo)

1. Sequencing, annotation, database development
2. Comparisons of species and strains
3. Proteomics
4. Glycomics

D. Basic biology of life-cycle stages

1. As themselves
2.
  - Male–female interactions
  - Female reproductive development and fecundity
3. As model systems
4.
  - Life-cycle stage shifts as developmental biology
  - Establishment of laboratory life cycles of *S. haematobium*
  - Expanded studies on experimental *S. japonicum*
5. Fecundity and egg excretion
6. Investigation of schistosome germ cells
7. Host–parasite interactions
8.
  - Role of host molecules in parasite development and life cycle
  - Identification of parasite molecules that regulate host function
9. Neurobiology and neuromuscular physiology

E. Biochemistry and molecular studies

1. Membrane biology
2. Metabolism using genomics, glycomics, and proteomics
3. Characterization and functional roles and uses of schistosome components
4. Development of “molecular tool box” for schistosomes
5.
  - Schistosome cell lines
  - RNAi and other gene silencing tools
  - Transient and stable transgenic schistosome cells or parasites
  - Expression of schistosome proteins in other eukaryotic systems
6. Important functional genes
7.
  - Isolate/investigate individual schistosome organs (e.g., ovary) or cells
  - Factors dictating host specificity in vertebrates and snails

Beyond the possibility of being used to elicit funding for schistosomiasis research, we hope that the process of compiling the agenda itself will serve to unite the community. At a minimum, a functioning list of about 350 e-mail addresses of people involved in schistosomiasis research and practice has been generated through this process. Hopefully, interactions within this group will lead to: (1) more schistosomiasis-focused interdisciplinary networks; (2) the development of standardized protocols for multicenter studies; (3) a higher profile for schistosomiasis within the global health community; (4) the further use of repositories of schistosome-related materials (such as http://www.schisto-resource.org/ and http://www.afbr-bri.com/sr3/); (5) recruitment of trainees; (6) enhanced mentoring of junior schistosome researchers; and (7) assistance in enlisting outside experts into the field of schistosomiasis (Image 2).

**Figure:**
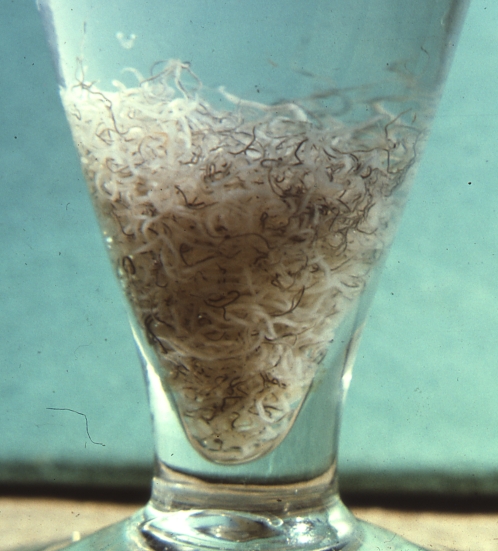
1659 adult *Schistosoma mansoni* worms obtained by live surgical perfusion of an 18 year-old patient in 1970

**Figure:**
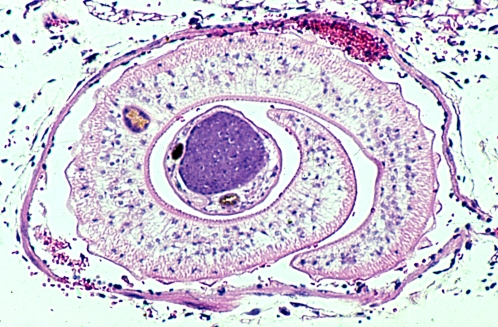
Cross-section of a *Schistosoma mansoni* adult worm pair in the mesenteric venule of a mouse; H&E stain

## Conclusion

This schistosomiasis research agenda resulted from querying of about 350 investigators and officials who care deeply about schistosomiasis. It reflects the breadth and depth of their perspectives on what is worth doing or finding out about schistosomes, their hosts, and how they interact. The agenda spans topics from social science to genomics. The true purpose of the agenda, and the process leading up to it, is not to debate whether one perspective is more important than another, but to help organize the schistosome community to move forward together. Through such discussions and collaborations, we hope to maximize the available resources (people, funds, field sites, outside experts, data sharing) and eventually better publicize the need for all research on this important disease, which in addition to advancing global public health efforts also has much to offer to fundamental biomedical knowledge. In addition, we hope that this will be an inclusive and living agenda. To make the latter attribute come true we invite readers of *PLoS Neglected Tropical Diseases* to annotate this preamble and the agenda itself, to start discussion threads based on individual components of the agenda, and to submit electronic letters to the editor concerning various aspects of the agenda. In addition, through the auspices of *PLoS ONE* and the community portals it will offer by next year, we hope that the agenda will serve as one focal point for interactive interchange among the schistosomiasis community, and thus provide a foundation for true collaborations within and across the spectrum of research to control of schistosomiasis.
